# Simplified Antenna Group Determination of RS Overhead Reduced Massive MIMO for Wireless Sensor Networks

**DOI:** 10.3390/s18010084

**Published:** 2017-12-29

**Authors:** Byung Moo Lee

**Affiliations:** School of Intelligent Mechatronics Engineering, Sejong University, Seoul 05006, Korea; blee@sejong.ac.kr; Tel.: +82-2-6935-2520

**Keywords:** antenna group, massive MIMO, reference signal (RS)

## Abstract

Massive multiple-input multiple-output (MIMO) systems can be applied to support numerous internet of things (IoT) devices using its excessive amount of transmitter (TX) antennas. However, one of the big obstacles for the realization of the massive MIMO system is the overhead of reference signal (RS), because the number of RS is proportional to the number of TX antennas and/or related user equipments (UEs). It has been already reported that antenna group-based RS overhead reduction can be very effective to the efficient operation of massive MIMO, but the method of deciding the number of antennas needed in each group is at question. In this paper, we propose a simplified determination scheme of the number of antennas needed in each group for RS overhead reduced massive MIMO to support many IoT devices. Supporting many distributed IoT devices is a framework to configure wireless sensor networks. Our contribution can be divided into two parts. First, we derive simple closed-form approximations of the achievable spectral efficiency (SE) by using zero-forcing (ZF) and matched filtering (MF) precoding for the RS overhead reduced massive MIMO systems with channel estimation error. The closed-form approximations include a channel error factor that can be adjusted according to the method of the channel estimation. Second, based on the closed-form approximation, we present an efficient algorithm determining the number of antennas needed in each group for the group-based RS overhead reduction scheme. The algorithm depends on the exact inverse functions of the derived closed-form approximations of SE. It is verified with theoretical analysis and simulation that the proposed algorithm works well, and thus can be used as an important tool for massive MIMO systems to support many distributed IoT devices.

## 1. Introduction

Massive multiple-input multiple-output (MIMO) system is a powerful technology that can increase both spectral efficiency (SE) and energy efficiency (EE), and it has been actively discussed to be included in the 3rd generation partnership project (3GPP) standard as core technology for 5G systems [1,2,3]. The massive MIMO system uses a large amount of transmitter (TX) antennas and serves limited number of user equipments (UEs) and/or Internet of things (IoT) devices/sensors, so it is a combination scheme of multi-user (MU) MIMO and beamforming, and its drastic performance gain has already been proven in various literature [4,5,6,7,8]. Recently, it has also been proven that massive MIMO is quite effective in supporting many Internet of things (IoT) and/or Industrial Internet of things (IIoT) devices, and thus can be used for a core technology to configure wireless sensor networks [9,10,11,12,13,14,15]. The calibration of transceiver impairment is very important problem to support distributed IoT devices using massive MIMO [9,10], because without getting accurate channel information, it is impossible to support the distributed IoT devices. In [9], authors proposed very effective channel calibration method to reduce the channel mismatch due to the radio frequency (RF) impairment. In their method, they used power headroom which is reported periodically and/or aperiodically from distributed IoT devices to base station (BS), to deliver the RF impairment information of IoT devices. Based on the RF impairment information which is embedded in power headroom, BS performs precoding with the compensation of channel mismatch. This is very important scheme to support distributed IoT devices using massive MIMO equipped data center. A more general work for transceiver impairment calibration was also proposed in [10]. In [10], authors investigated massive MIMO-based distributed detection with general transceiver impairments at both a massive-antenna data center and multiple single-antenna sensors. They first derived closed-form expressions to show the dependence of both the probability of detection and the probability of false alarm on the transceiver impairments, then they showed that hardware impairments create finite ceilings on the detection performance. In addition, they formulated an optimization problem to maximize the probability of detection, while maintaining a constant false alarm probability and a sum sensor reporting power budget. By exploiting the inherent structures in the problem formulation, they developed an iterative algorithm to solve the problem via invoking the alternating direction method of multipliers. In addition with the results, there has been a high interest to equip the sensor fusion center with massive MIMO framework [11,12,13,14,15]. In [11], authors considered a decentralized multi-sensor estimation problem where sensor nodes observe noisy versions of a correlated random source vector. The sensors amplify and forward their observations over a fading coherent multiple access channel to a fusion center. The fusion center is equipped with a massive MIMO and adopts a minimum mean-square error (MMSE) approach for estimating the source. They optimized the transmission power at each sensor node to increase energy efficiency in various scenarios. In [12,13], authors studied channel-aware decision fusion over MIMO channels, in the presence of a massive MIMO at the fusion center. They tried to develop linear fusion rules, and presented a wide choice of low-complexity sub-optimal rules which efficiently exploit massive MIMO benefits and are able to achieve near-optimal performance. In [14], authors considered the uplink detection and estimation of a zero mean Gaussian signal in a wireless sensor network when the fusion center is configured with a massive MIMO. For the detection problem, they studied the Neyman-Pearson (NP) detector and energy detector (ED), and found optimal values for the sensor transmission gains. According to the results, while bounds derived for NP detection shows performance gains for a fusion center with massive MIMO in low sensor transmit power scenarios, the benefit is shown to disappear when the transmit power is high. However, for the ED, having massive MIMO at the fusion center provides a significant advantage even when the sensors have high power. In [15], authors considered distributed detection in wireless sensor networks with a massive MIMO fusion center. Using the large deviation principle and random matrix theory, they analyzed the asymptotic detection performance of optimal hypothesis testing in terms of error exponents for the false alarm and miss detection probabilities which enable us to predict how difficult it will be to attain a certain level of detection reliability.

Various studies already have shown that massive MIMO is quite effective to support distributed IoT devices. However, one of big obstacles to the realization of massive MIMO systems is reference signal (RS) overhead, which increases as the number of transmitter (TX) antennas and/or user entities (UEs) increases. The RS overhead reduction is a classical issue in research field of wireless communication, and numerous related technologies have been introduced [16,17,18,19,20]. In general, for massive MIMO, it is very difficult to apply orthogonal RS to each antenna due to the excessive amount of TX antennas, thus antenna group-based RS overhead reduction scheme is generally applied [20,21]. In this kind of scheme, in each antenna group, the same RS is shared. In the case, there is enough correlation among antennas, antenna group can be decided based on beam groups and locations of distributed UEs. If there is not enough correlation in each antenna, several RS superposition schemes can be applied. A blind channel estimation scheme using the specific statistical property of the signal and the channel were introduced in [22,23]. A training based super-imposed signal, which locates the RS stream and data stream in the same resources, and can also be applied to super-imposed RS streams, were introduced in [24,25]. It is already proven that the antenna group-based RS overhead reduction scheme is quite effective. One of important factors in designing the antenna group-based RS overhead reduction scheme is how many antennas will be grouped in each group. Several techniques that can effectively be applied to antenna group-based RS overhead reduction scheme have been proposed [20,21], while little work has been done for the selection criterion of the number of antennas needed in each group, and related system design [16,17,18,19,22,23,24,25]. All of the work described above focuses solely on the methodology of how to reduce RS overhead. In real situation, if we assume we have the RS overhead reduction scheme at hand based on any kind of existing methodology, before applying the scheme, it is necessary to decide the target amount of RS overhead reduction in given situations. In addition, it is also necessary to design the systematic operational step and apparatus for real implementation. As far as we know, these have not been actively discussed in the literature yet.

In this paper, we propose a simplified determination scheme of the number of antennas needed in each group for massive MIMO to support wireless sensor networks, and present related system structure for the operation. Basically, our contribution in this paper can be divided into two parts. First, we analyze the performance of RS overhead reduced massive MIMO systems with antenna grouping and related channel estimation error. Even though antenna grouping-based RS overhead reduction scheme is quite effective for the operation of massive MIMO and related IoT device support, channel estimation error is inevitable. The closed-form approximations of achievable SE, which includes related channel estimation error factor, are derived. Based on the channel estimation error factor, both RS overhead performance and the seriousness of channel estimation error can be adjusted. The seriousness of channel estimation error which is used in this paper will be shown in Section 5. We also show that the derived closed-form approximations are quite simple and effective, and in good agreement with the simulation results. Second, based on the derived closed-form approximation of SE, and using the exact inverse of the SE closed-form approximation, we propose a simplified determination scheme of the number of antennas needed in each group. Without this scheme, it is very difficult to apply antenna grouping-based RS overhead reduction scheme in a real system. The change of parameters must be reflected in real time to effectively determine the number of antennas in each group. The presented system block diagram and flow chart can be greatly helpful to real implementation of the proposed scheme. In a word, based on closed-form approximation of SE, to support distributed IoT devices, we propose a simplified determination scheme of the number of antennas needed in each group. More specifically, our main contributions are summarized as follows:

- We present the closed-form approximations of achievable SE with channel estimation error for two representative linear precodings. We used the error factor which reflects the degree of channel estimation error and the error matrix which has the same statistical characteristic but independent of the channel matrix. The range of error factor is between 0 and 1, and it is a function of the number of antennas in each antenna group, and the adjustable factor which reflects the seriousness of the channel estimation error depending on the applied reference signal (RS) overhead reduction technique. We prove the derived SEs are quite simple and effective using extensive simulation results.
- Based on the closed-form approximations of achievable SEs, we derive the determination criteria of the number of antennas needed in each antenna group. The determination criteria are derived from the inverse functions of achievable SEs.
- We provide the system block diagram and algorithmic steps to apply the proposed determination scheme. The system block diagram is consisted of 5 main blocks, The central management unit, operation parameters unit, minimum data rate unit, decision unit, and UE. There are 3 thresholds we should consider for the systematic operation, i.e., power consumption, data rate, and the number of iterations.
- We provide the extensive simulation results of the proposed scheme with various parameters, and show that the derived approximations are very effective and well-matched with the simulation results.

The paper is organised as follows: the system description is presented in Section 2. Massive MIMO model, related precoding techniques, and RS overhead model are given in Section 2. In Section 3, the performance analysis and closed-forms approximation of achievable SE are presented. In Section 4, the determination scheme of the number of antennas in each group is shown. In Section 5, the proposed model is numerically analyzed. With monte-carlo (MC) simulations, we show that the analysis is well-matched with simulation results. Finally, concluding remarks are given in Section 6.

*Notation*: In the rest of the paper, boldface lower-case and upper-case characters denote vectors and matrices, respectively. The operators ${\left(\cdot \right)}^{H}$ and E[·] denote conjugate transpose and expectation, respectively. The N×N identity matrix is denoted $I_{N}$, and the N×N zero matrix is denoted $0_{N}$. $X∼N(0_{N},\;\;V_{N})$ is the complex Gaussian distributed vector with mean zero and covariance $V_{N}$. $\log_{2}\left(\cdot \right)$ denotes the common logarithm and ${∥\cdot ∥}_{F}$ represents the Frobenius norm operator. We use ∘ to denote the componentwise product of the matrices. we use $diag\left[d_{1},\cdots ,d_{N}\right]$ for N×N diagonal matrix with $d_{i}$ as the *i*th diagonal element.

## 2. System Description

### 2.1. Massive MIMO Model

We consider a downlink massive MIMO system with $N_{t}$ TX antennas, and *K* single antenna UEs and/or IoT devices/sensors. The received signal vector at UEs can be represented as follows:

(1) $$\begin{array}{ccc} y & = & \sqrt{\rho_{t}}Gs+n, \end{array}$$

where y is the K×1 received signal vector for *K* UEs, $\rho_{t}$ is the total TX power for forward link, G is the $K\times N_{t}$ channel matrix between the transmitter with $N_{t}$ TX antennas and *K* UEs, s is the $N_{t}\times 1$ TX signal vector, and n is the K×1 additive white Gaussian noise (AWGN) vector at the UEs (i.e., $n∼CN(0_{K}^{},\sigma_{d}^{2}I_{K}^{})$) where $\sigma_{d}^{2}$ is the variance of AWGN. G consists of both a small scale fading channel matrix, H and large scale fading channel matrix, B, i.e., G=H∘B where ∘ indicates the componentwise product. H is the independent and identically distributed (i.i.d) Rayleigh fading channel matrix with zero mean and unit variance which can be represented as:

(2) $$H=\left(\begin{array}{cccc} h_{1,1} & h_{1,2} & \cdots & h_{1,N_{t}}\\ h_{2,1} & h_{2,2} & \cdots & h_{2,N_{t}}\\ \vdots & \vdots & \vdots & \vdots \\ h_{K,1} & h_{K,2} & \cdots & h_{K,N_{t}} \end{array}\right),$$

B is the large scale fading channel matrix which can be represented as:

(3) $$B=\left(\begin{array}{cccc} \sqrt{\beta_{1,1}} & \sqrt{\beta_{1,2}} & \cdots & \sqrt{\beta_{1,N_{t}}}\\ \sqrt{\beta_{2,1}} & \sqrt{\beta_{2,2}} & \cdots & \sqrt{\beta_{2,N_{t}}}\\ \vdots & \vdots & \vdots & \vdots \\ \sqrt{\beta_{K,1}} & \sqrt{\beta_{K,2}} & \cdots & \sqrt{\beta_{K,N_{t}}} \end{array}\right),$$

where $\beta_{i,j}$ is the path loss component from *j*th antennas in BS to *i*th UE. Then, the combined channel matrix G=H∘B can be represented as:

(4) $$G=\left(\begin{array}{cccc} h_{1,1}\sqrt{\beta_{1,1}} & h_{1,2}\sqrt{\beta_{1,2}} & \cdots & h_{1,N_{t}}\sqrt{\beta_{1,N_{t}}}\\ h_{2,1}\sqrt{\beta_{2,1}} & h_{2,2}\sqrt{\beta_{2,2}} & \cdots & h_{2,N_{t}}\sqrt{\beta_{2,N_{t}}}\\ \vdots & \vdots & \vdots & \vdots \\ h_{K,1}\sqrt{\beta_{K,1}} & h_{K,2}\sqrt{\beta_{K,2}} & \cdots & h_{K,N_{t}}\sqrt{\beta_{K,N_{t}}} \end{array}\right).$$

Since the path loss components from 1th, 2nd, ⋯, $N_{t}$th antenna in BS to antenna in *i*th UE are almost the same, for the path loss component from BS to *i*th UE, $\beta_{i}$, we can represent as follows:

(5) $$\beta_{i}=\beta_{i,1}=\beta_{i,2},\cdots ,\beta_{i,N_{t}}.$$

where i=1,2,⋯,K.

Note that, to get the benefit of channel hardening effect, usually the massive MIMO antenna systems should satisfy the condition of $N_{t}>10K$ [26,27]. There is no strict criterion to get the channel hardening effect, but we can asymptotically observe the effect when $N_{t}$ is larger than 10K.

**Figure 1:**
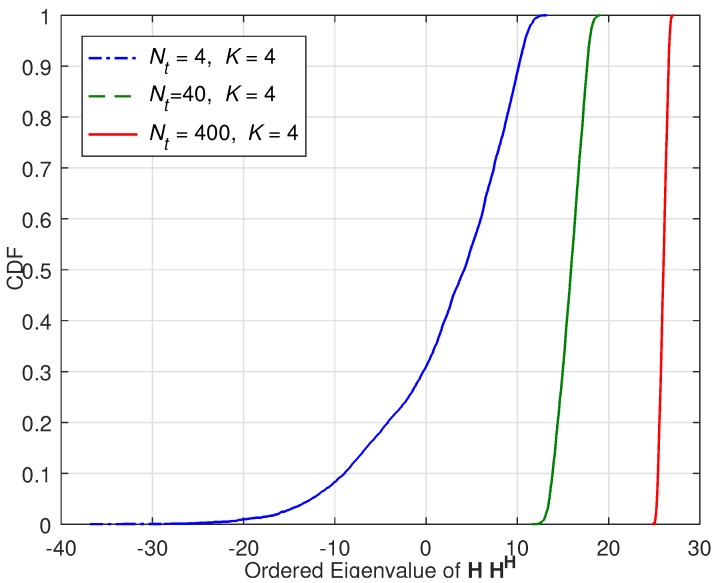
Example of channel hardening effect, when $N_{t}=4,\;40,\;400$ and K=4, CDF: cumulative distribution function.

**Figure 2:**
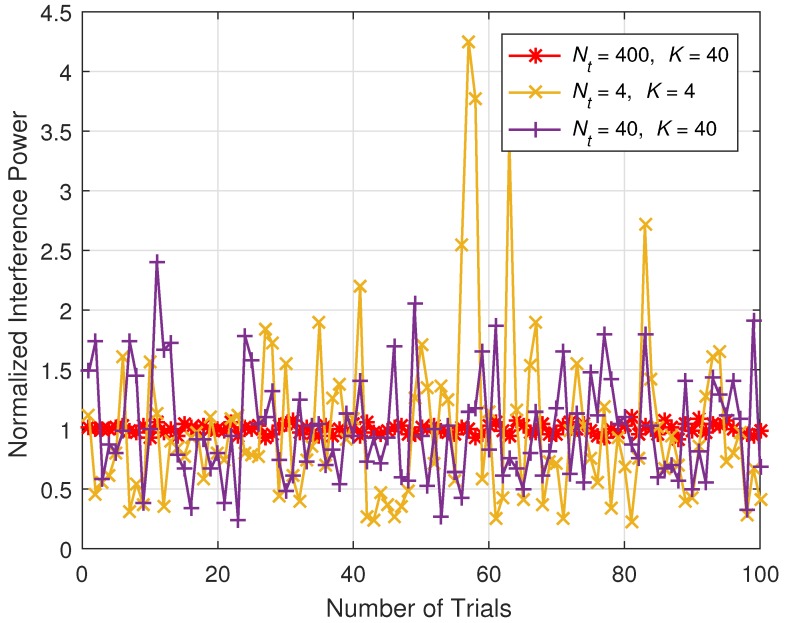
Normalized Interference Power versus Number of Trials: As $N_{t}$ increases, the interference level converges to a certain point that can be easily estimated.

Figure 1 presents the simulation to show the channel hardening effect. The x-axis of the figure represents the ordered eigenvalue of $HH^{H}$, and y-axis represents the cumulative distribution function (CDF). The figure shows the case in which the number of UEs is fix to K=4, and the number of TX antennas increases as $N_{t}=4,\;40$, and 400. As $N_{t}$ increases, the randomness of the channel significantly reduces. Likewise, due to the law of large numbers, as $N_{t}$ increases, the level of interference also converges to a certain constant. Figure 2 presents the normalized interference power versus number of trials. Figure 2 is the result when we use the matched filtering (MF) precoding which will be introduced in the next subsection. As observed, as $N_{t}$ increases, the variation of interference reduces. It is very close to a constant when $N_{t}=10K$. From Figure 1 and Figure 2, we can observe that asymptotically the channel hardening effect is reliable enough when $N_{t}>10K$, but as we mentioned, this is just asymptotic approach and there is not strict rule for the criterion of channel hardening effect.

### 2.2. Precoding

Since $N_{t}$ is much larger than *K*, we should map *K* message signal to each antenna. In general, we call it precoding, and it is applied to the transmitter part to reduce inter-user interference (IUI). Since $N_{t}$ is very large, the linear precoding should be used for real systems [28,29,30]. For regular MIMO systems, both nonlinear and linear precoding scheme could be considered. Nonlinear precoding techniques, such as dirty paper coding (DPC) [31] , vector perturbation (VP) [32], and lattice-aided methods [33] are important techniques when $N_{t}$ is not much larger than *K*. However, with an increase in the number of antennas at the BS, linear precoders are shown to be near-optimal [28,29]. When $N_{t}$ is, say, two times *K*, this gap is only 3 dB [29]. It is shown that with linear precoding, a sum rate as high as 98% of that of the DPC scheme can be achieved for two single antenna users served by 20 BS antennas [30]. Thus, it is more practical to use low-complexity linear precoding techniques in massive MIMO systems. Therefore, we mainly focus on linear precoding techniques.

It is well-known in the literature that zero-forcing (ZF) and regularized zero-forcing (RZF) are effective linear precoding techniques [34]. Also, in [4], T. Marzetta suggested even a simpler precoding technique, matched filtering (MF) precoding. The TX signal vector s and the message signal vector x are related as follows:

(6) s=ζFx,

where ζ is the normalization factor of TX power and F is a precoding matrix. Then, (1) can be represented as follows:

(7) $$\begin{array}{ccc} y & = & \sqrt{\rho_{t}}G\zeta Fx+n, \end{array}$$

where F is the $N_{t}\times K$ precoding matrix. The three representative precoding matrices are denoted in Table 1.

MF precoding matrix is simply the conjugate transpose of the channel matrix. This is conceptually simplest approach to reduce the IUI. In MF precoding, it is seen that the signal-to-interference and noise ratio (SINR) can be made as high as desired by increasing the number of antennas. However, the MF precoding exhibits an error floor in the practical number of TX antennas. The main advantage of MF precoding is its low computational complexity. Another advantage is that it is effective for distributed antenna systems, because the Massive MIMO signal processing can be performed locally at each antenna [5]. Another approach for precoding is to invert the channel by means of the pseudo-inverse. This is referred to as ZF precoding. When $N_{t}>K$, ZF precoding completely removes the IUI. Moreover, when SNR is high and/or $N_{t}\gg K$, ZF precoding can achieve nearly optimum performance [29]. However, a disadvantage of ZF is that processing cannot be done distributedly at each antenna separately. With ZF precoding, all data must instead be collected at a central node that handles the processing [29]. MF precoding can outperform ZF precoding when SNR is very low, while ZF precoding outperform MF precoding when SNR is relatively high. When SNR is not so high, ZF precoding cannot give any meaningful performance. RZF precoding can combat this problem. When SNR is high, both ZF precoding and RZF precoding shows very similar performance, while when SNR is low, RZF precoding outperforms ZF precoding. When SNR is very low, RZF prcoding show similar performance with MF precoding. A brief summary of the advantages and disadvantages of the MF, ZF, and RZF is shown in Table II of [30].

The normalization factor should be determined such that the total transmit power becomes $\rho_{t}$. It can be expressed as follows:

(8) $${∥\zeta Fx∥}_{F}^{2}=1,$$

where ${∥∥}_{F}$ stands for Frobenius norm.

ζ for *k*th UE, $\zeta_{k}$ is approximated as $\zeta_{k,mf}\approx \frac{1}{\sqrt{N_{t}K}}$ for MF precoding and $\zeta_{k,ZF}\approx \sqrt{\frac{N_{t}-K}{K}}$ for ZF precoding [35].

Both ZF and RZF precodings show very similar performance, even in the relatively practical number of massive TX antennas (i.e., $N_{t}\approx 10K$ ).

### 2.3. RS Overhead Model

As a reference model, the current 3GPP LTE systems use following types of RSs; common reference signal (CRS), channel state information reference signal (CSI-RS), demodulation reference signal (DM-RS), multicast-broadcast single-frequency network (MBSFN) reference signal, positioning reference signal (PRS). In this paper, we only consider CRS, CSI-RS, and DM-RS, because the three signals take most of resources for RS [36,37]. PRS are only transmitted in resource blocks over downlink subframes that are configured for PRS transmission, and The MBSFN reference signals are only transmitted in the MBSFN region of MBSFN subframes [36,37], thus PRS and MBSFN are not included in general operation mode and we disregard both cases for simplicity.

CRS is called as a cell specific reference signal, and has been in the LTE system from Release 8. The role of CRS can be defined as the cell search and initial acquisition, the downlink channel estimation for coherent demodulation/detection at the UE, and the downlink channel quality measurements. CSI-RS has been introduced from Release 10, and used by UE to estimate the channel and report channel quality information (CQI) to BS. DM-RS is usually called as an UE specific reference signal, and the role of it is for the demodulation of the signal.

In this paper, assuming frequency division multiplexing (FDD) mode, we use the following RS overhead factor for analysis [20]:

(9) $$\begin{array}{ccc} \chi_{}\left(\%\right) & = & \left(\eta_{CRS}+N_{t}+K\cdot \psi_{DM-RS}\right)/\eta_{RB_{tot}}\times 100, \end{array}$$

where $\chi_{}\left(\%\right)$ is expressed as a percentage of RS overhead in a given total resources, $\eta_{CRS}$ is the number of CRS in a given resource, $\psi_{DM-RS}$ is the DM-RS proportional factor of *K* which we use 10, and $\eta_{RB_{tot}}$ is the total resource elements for a given resource block for a coherence time. In the current 3GPP LTE-A systems, the number of resource elements available in two resource blocks (1 ms ) is 168 (12 (frequency tones) × 14 (time symbols)). Then, $\eta_{RB_{tot}}=840$ resource elements assuming 5 ms coherence time. The CRS takes 14.8% of available resource elements, which is not a small portion. We do not know how technology will be evolved from an RS perspective. However it is quite obvious that RS overhead increases as the number of TX antennas increases. In this respect, (9) is reliable enough to use. The expected RS overhead reduction factor can be written as follows [20]:

(10) $$\begin{array}{ccc} \chi_{r}\left(\%\right) & = & \left(\eta_{CRS}+\left(N_{t}+K\cdot \psi_{DM-RS}\right)/N_{g}\right)/\eta_{RB_{tot}}\times 100, \end{array}$$

where $N_{g}$ is the RS overhead reduction factor to represent the number of antennas in each group, and the performance of RS overhead reduction scheme. Here we assume that the CRS is irreducible for cell search and initial acquisition, downlink channel estimation for coherent demodulation/detection at the UE, and downlink channel quality measurements, while all of the other RSs are ideally reducible by the RS overhead reduction scheme. Since resources of the CSI-RS and DM-RS are shared in the same group of antenna elements, it is divided by $N_{g}$. In other word, $N_{g}$ number of antenna elements share the same CSI-RS and DM-RS resources, and they are not orthogonal in each group, thus in each group, CSI-RS and DM-RS resources are reduced by factor of $N_{g}$, but CRS is irreducible and not divided by $N_{g}$, because it requires high accuracy. Reducing RS resources causes channel estimation error.

## 3. Closed-Form Approximation of Achievable Spectral Efficiency with Channel Estimation Error

In this Section, we present the closed-form approximation of achievable SE with channel estimation error. We should keep in mind that antenna grouping-based RS overhead reduction scheme causes channel estimation error. As $N_{g}$ increases, channel estimation error increases. The estimated channel, $\hat{H}$, is modeled as follows:

(11) $$\hat{H}=\xi H+\sqrt{1-\xi^{2}}E,$$

where ξ∈[0,1] is the error factor, which reflects the degree of channel estimation error, and $E\in C^{K\times N_{t}}$ is the error matrix with the same statistical characteristic but independent of the channel, H. ξ can also be modeled as follows:

(12) $$\xi =\sqrt{\hat{\xi }\left(1-\frac{N_{g}}{\varepsilon }\right)},$$

where $\hat{\xi }$ is the channel estimation error regardless of antenna group, and ε is the adjustable factor to reflect the seriousness of the channel estimation error depending on the applied technique.

For the performance analysis, the maximum achievable SE can be derived by using i.i.d. Rayleigh channel with zero mean and unit variance. From (1), and (7), the symbol received by *k*-th user is given by

(13) $$y_{k}=\sqrt{\rho_{t}}\zeta_{k}\left(h_{k}\circ b_{k}\right)f_{k}x_{k}+n_{k}+\sqrt{\rho_{t}}\zeta_{l}\sum_{l\neq k}\left(h_{k}\circ b_{k}\right)f_{l}x_{l},$$

where $h_{k}$ and $b_{k}$ are the $1\times N_{t}$ channel vectors for *k*-th user, and $f_{k}$ is the $N_{t}\times 1$ precoding vector for *k*-th UE. The last term of (13) is the IUI. $\zeta_{k}$ is a normalized factor for the precoding process of *k*-th UE, and it is approximated as $\zeta_{k,mf}\approx \frac{1}{\sqrt{N_{t}K}}$ for MF precoding and $\zeta_{k,ZF}\approx \sqrt{\frac{N_{t}-K}{K}}$ for ZF precoding [35] as we mentioned in the previous section. Assuming all the path loss component to UE *k* is the same, which is true in a real system, the effective SINR at the receiver (RX) for user *k*, $\gamma_{k}$, can be expressed as follows:

(14) $$\gamma_{k}=\frac{\rho_{r}{\left|\zeta_{k}h_{k}f_{k}\right|}^{2}}{\rho_{r}{\left|\sum_{l\neq k}\zeta_{l}h_{k}f_{l}\right|}^{2}+1},$$

where $\rho_{r}=\frac{\rho_{t}\cdot \beta_{k}}{N_{0}B}$ is the received signal-to-noise ratio at RX, $\beta_{k}$ is the large scale fading or path loss component between TX and *k*th UE, and $N_{0}B$ is the noise power in the given bandwidth, *B*. As was shown in the previous section, path loss components from 1st, 2nd, 3rd, ⋯, $N_{t}$th antenna in BS to antenna in *k*th UE are the same ($\beta_{i}=\beta_{i,1}=\beta_{i,2},\cdots ,\beta_{i,N_{t}}$). This is because the distances among antennas in BS are much shorter than the distances between antennas in BS and antennas in UEs.

The data rate, *R* of single isolated cell can be represented as follows:

(15) $$R=\alpha \cdot B\cdot \sum_{k=1}^{K}E\left[\log_{2}\left(1+\gamma_{k}\right)\right],$$

where α is the scaling factor for RS overhead [4], and E[·] is the expectation operation. In the ideal case, i.e., perfect blind channel estimation with no channel estimation error, α is equal to 1. In a real situation, however, by using (10), we can say that $\alpha =\left(1-\frac{\chi_{r_{}}}{100}\right)$, when a RS overhead reduction scheme is applied. Since the scaling factor should be more than 0.5 in the system design perspective, the spectral efficiency (SE) based on capacity analysis can be represented as follows:

(16) $$SE_{}\approx \left\{\begin{array}{c} \alpha \cdot \sum_{k=1}^{K}E\left[\log_{2}\left(1+\gamma_{k}\right)\right],\;\;\alpha >0.5.\\ 0.5\cdot \sum_{k=1}^{K^{\prime }}E\left[\log_{2}\left(1+\gamma {{}^{\prime }}_{k}\right)\right],\;\;\alpha \leq 0.5. \end{array}\right.$$

where $K{}^{\prime }$ and $\gamma {{}^{\prime }}_{k}$ represent corresponding constant when overhead is 0.5.

We show the reference SINR based on MF precoding. By using channel hardening effect of the massive MIMO systems [4], the reference SINR based on MF precoding, $\gamma_{k,mf}^{ref}$, can be simplified as follows [38]:

(17) $$\gamma_{k,mf}^{ref}=\frac{\rho_{r}{\left|\zeta_{k,mf}h_{k}h_{k}^{H}\right|}^{2}}{\rho_{r}\sum_{l\neq k}{\left|\zeta_{l,mf}h_{k}h_{l}^{H}\right|}^{2}+1}\to \frac{N_{t}}{K}\left(\frac{\rho_{r}}{I_{mf}+1}\right)=\frac{N_{t}}{K}\left(\frac{\rho_{r}}{\rho_{r}+1}\right),$$

where $I_{mf}$ is the IUI term after MF precoding. For massive MIMO region, $I_{mf}$ can be simplified to $\rho_{r}$ [4]. Here we can say that $\frac{Nt}{K}$ is the channel gain of the massive MIMO system.

It is obvious that if we increase the number of TX antennas or reduce the number of UEs, we can get a more effective SINR. However, it should be noted that MF precoding does not completely remove the IUI in practical number of TX antennas. It is known that ZF precoding can completely remove IUI with the perfect channel information, but channel gain is reduced to $N_{t}-K$ as compared with MF precoding, $N_{t}$.

When there is a channel estimation error, the effective SINR based on MF precoding can be represented as follows:

(18) $${\hat{\gamma }}_{k,mf}=\frac{\rho_{r}{\left|\zeta_{k,mf}h_{k}^{}{\hat{h}}_{k}^{H}\right|}^{2}}{\rho_{r}\sum_{l\neq k}{\left|\zeta_{l,mf}h_{k}^{}{\hat{h}}_{l}^{H}\right|}^{2}+1},$$

where ${\hat{h}}_{k}^{}$ is the $1\times N_{t}$ estimated channel vector for *k*-th UE. Using (11) and statistical approximation, (18) can be simplified as follows:

(19) $$\begin{array}{cc} {\hat{\gamma }}_{k,mf} & =\frac{\rho_{r}{\left|\zeta_{k,mf}\xi h_{k}^{}h_{k}^{H}+\zeta_{k,mf}\sqrt{1-\xi^{2}}h_{k}^{}e_{k}^{H}\right|}^{2}}{\rho_{r}\sum_{l\neq k}{\left|\zeta_{l,mf}\xi h_{k}^{}h_{l}^{H}+\zeta_{l,mf}\sqrt{1-\xi^{2}}h_{k}^{}e_{l}^{H}\right|}^{2}+1},\\ & \to \frac{N_{t}}{K}\left(\frac{\xi_{}^{2}\rho_{r}}{\rho_{r}+1}\right), \end{array}$$

where $e_{l}$ is the $1\times N_{t}$ channel estimation error vector. For Equation (19), we use the following approximations:

(20) $${\left|\zeta_{k,mf}\xi h_{k}^{}h_{k}^{H}+\zeta_{k,mf}\sqrt{1-\xi^{2}}h_{k}^{}e_{k}^{H}\right|}^{2}\approx \xi_{}^{2}{\left|\zeta_{k,mf}h_{k}^{}h_{k}^{H}\right|}^{2},$$

(21) $$\sum_{l\neq k}{\left|\zeta_{k,mf}\xi h_{k}^{}h_{l}^{H}+\zeta_{k,mf}\sqrt{1-\xi^{2}}h_{k}^{}e_{l}^{H}\right|}^{2}\approx \sum_{l\neq k}{\left|\zeta_{k,mf}h_{k}^{}h_{l}^{H}\right|}^{2}.$$

The approximation of (20) and (21) comes from the following equations:

(22) $${\left|\zeta_{k,mf}\xi h_{k}^{}h_{k}^{H}\right|}^{2}\gg {\left|\zeta_{k,mf}\sqrt{1-\xi^{2}}h_{k}^{}e_{k}^{H}\right|}^{2},$$

(23) $$\sum_{l\neq k}{\left|\zeta_{k,mf}\xi h_{k}^{}h_{l}^{H}+\zeta_{k,mf}\sqrt{1-\xi^{2}}h_{k}^{}e_{l}^{H}\right|}^{2}\approx \sum_{l\neq k}{\left|\zeta_{k,mf}\xi h_{k}^{}h_{l}^{H}+\zeta_{k,mf}\sqrt{1-\xi^{2}}h_{k}^{}h_{l}^{H}\right|}^{2}.$$

By comparing (17) and (19), it can be seen that the SINR based on MF precoding with channel estimation error has the same characteristics, but the signal power is scaled down by the square of the error factor, ξ.

Using (11) and statistical approximation, The effective SINR based on ZF precoding with channel estimation error can be written as follows [39]:

(24) $$\begin{array}{cc} {\hat{\gamma }}_{k,ZF} & =\frac{{\left|\zeta_{k,ZF}h_{k}^{}{\left(\hat{H}{\hat{H}}^{H}\right)}^{-1}{\hat{h}}_{k}^{H}\right|}^{2}}{h_{k}^{}{\left(\hat{H}{\hat{H}}^{H}\right)}^{-1}{\hat{H}}_{\left[k\right]}{\hat{H}}_{\left[k\right]}^{H}{\left(\hat{H}{\hat{H}}^{H}\right)}^{-1}h_{k}^{H}\sum_{l\neq k}\zeta_{l,ZF}+1},\\ & \to \frac{N_{t}-K}{K}\left(\frac{\xi^{2}\rho_{r}}{\left(1-\xi^{2}\right)\rho_{r}+1}\right), \end{array}$$

where ${\hat{H}}_{\left[k\right]}^{H}=\left[{\hat{h}}_{1}^{H},{\hat{h}}_{2}^{H},\cdots ,{\hat{h}}_{k-1}^{H},{\hat{h}}_{k+1}^{H},\cdots ,{\hat{h}}_{K}^{H}\right]\in C_{}^{N_{t}\times \left(K-1\right)}$.

For (24), the following approximations are used:

(25) $$\begin{array}{c} {\left|\zeta_{k,ZF}h_{k}^{}{\left({\left(\xi H_{k}^{}+\sqrt{1-\xi^{2}}E_{k}^{}\right)}^{}{\left(\xi H_{k}^{}+\sqrt{1-\xi^{2}}E_{k}^{}\right)}^{H}\right)}^{-1}{\hat{h}}_{k}^{H}\right|}^{2}\\ \approx \xi^{2}{\left|\zeta_{k,ZF}h_{k}^{}h_{k}^{H}\right|}_{}^{2}, \end{array}$$

(26) $$\begin{array}{c} h_{k}{\left(\left(\xi H_{k}^{}+\sqrt{1-\xi^{2}}E_{k}^{}\right){\left(\xi H_{k}^{}+\sqrt{1-\xi^{2}}E_{k}^{}\right)}^{H}\right)}^{-1}\cdot \\ (\left(\xi H_{\left[k\right]}^{}+\sqrt{1-\xi^{2}}E_{\left[k\right]}^{}\right){\left(\xi H_{\left[k\right]}^{}+\sqrt{1-\xi^{2}}E_{\left[k\right]}^{}\right)}^{H})\cdot \\ {\left(\left(\xi H_{k}^{}+\sqrt{1-\xi^{2}}E_{k}^{}\right){\left(\xi H_{k}^{}+\sqrt{1-\xi^{2}}E_{k}^{}\right)}^{H}\right)}^{-1}\cdot h_{k}^{}\sum_{l\neq k}\zeta_{l,ZF}\\ \approx \left(1-\xi^{2}\right)\sum_{l\neq k}{\left|\zeta_{l,ZF}h_{k}^{}{\hat{h}}_{l}^{H}\right|}^{2}. \end{array}$$

From (17) and (24), we can see that the SINR based on ZF precoding with channel estimation error is scaled down by $\xi^{2}$ and $\left(1-\xi^{2}\right)$ for the signal and the interference power from reference SINR based on MF precoding, respectively. Obviously, the residual IUI term for ZF precoding is due to channel estimation error.

The SINRs for the cases of reference MF precoding, reference ZF precoding, MF precoding with channel estimation error, and ZF precoding with channel estimation error are summarized in Table 2.

If we put the SINR in the Table 2 into the Equation (16), we can get the theoretical achievable SE of RS overhead reduction scheme with channel estimation error. In a real situation, the channel estimation error due to any kind of interference can be reflected in error factor ξ. The RS overhead reduction performance can be also reflected in RS overhead reduction factor and/or the number of antenna in each group, $N_{g}$. $N_{g}$ increment guarantees $N_{t}$ increment. However, increasing $N_{g}$ is not always beneficial. As we can see from Equation (12), $N_{g}$ increment also causes ξ increment. Obviously, ξ increment can cause performance reduction.

In the case of RZF precoding, the SINR is shown in Equation (19) of [39] and Equation (17) of [40]. Substituting the SINR of RZF, $\gamma_{k,RZF}$ into Equation (16), one can obtain the achievable SE of RZF precoding. Since the expressions in [39,40] are too complex to use in real system analysis, we show the simplified expression using the SINRs of MF and ZF precoding. Basically in low power regime, the SINR of RZF precoding is similar to MF precoding. In high power regime, the SINR of RZF is similar to ZF precoding. Based on these facts, we can approximate the SINR of RZF with channel estimation error as follows:

(27) $${\hat{\gamma }}_{k,RZF}\approx \left\{\begin{array}{c} \frac{\left(N_{t}-K\right)\hat{\xi }\left(1-\frac{N_{g}}{\varepsilon }\right)\rho_{r}}{K\left(\left(1-\hat{\xi }\left(1-\frac{N_{g}}{\varepsilon }\right)\right)\rho_{r}+1\right)},\;\;\;\;\;\;\;\;N_{t}\rho_{r}\gg K,\;\;\\ \frac{N_{t}\hat{\xi }\left(1-\frac{N_{g}}{\varepsilon }\right)\rho_{r}}{K(\rho_{r}+1)},\;\;\;\;\;\;\;\;N_{t}\rho_{r}\ll K. \end{array}\right.$$

Since we assume the system with $N_{t}>10K$, due to the distinct channel gain, the SINR of RZF precoding is similar to the SINR of ZF precoding. We will show this in Section 5. Readers can refer to [39,40] for more details of RZF precoding.

## 4. $N_{g}$ Determination and Related System Structure

In this Section, we propose how to determine $N_{g}$ in an antenna group-based RS overhead reduced massive MIMO. Based on analysis in Section 3, ZF and MF precoded SE, $SE_{ZF}$ and $SE_{MF}$ can be approximated as follows:

(28) $$SE_{ZF}\approx \left\{\begin{array}{c} \left(1-\frac{\eta_{CRS}+\left(N_{t}+K\cdot \psi \right)}{\eta_{RB_{tot}}N_{g}}\right)\cdot K\cdot \left[\log_{2}\left(1+\frac{\left(N_{t}-K\right)\hat{\xi }\left(1-\frac{N_{g}}{\varepsilon }\right)\rho_{r}}{K\left(\left(1-\hat{\xi }\left(1-\frac{N_{g}}{\varepsilon }\right)\right)\rho_{r}+1\right)}\right)\right],\;\left(1-\frac{\eta_{CRS}+\left(N_{t}+K\cdot \psi \right)}{\eta_{RB_{tot}}N_{g}}\right)>0.5\\ 0.5\cdot K^{\prime }\cdot \left[\log_{2}\left(1+\frac{\left(N_{t}^{\prime }-K^{\prime }\right)\hat{\xi }\left(1-\frac{N_{g}^{\prime }}{\varepsilon }\right)\rho_{r}}{K\left(\left(1-\hat{\xi }\left(1-\frac{N_{g}^{\prime }}{\varepsilon }\right)\right)\rho_{r}+1\right)}\right)\right],\;\left(1-\frac{\eta_{CRS}+\left(N_{t}+K\cdot \psi \right)}{\eta_{RB_{tot}}N_{g}}\right)\leq 0.5 \end{array}\right.$$

(29) $$SE_{MF}\approx \left\{\begin{array}{c} \left(1-\frac{\eta_{CRS}+\left(N_{t}+K\cdot \psi \right)}{\eta_{RB_{tot}}N_{g}}\right)\cdot K\cdot \left[\log_{2}\left(1+\frac{N_{t}\hat{\xi }\left(1-\frac{N_{g}}{\varepsilon }\right)\rho_{r}}{K(\rho_{r}+1)}\right)\right],\;\left(1-\frac{\eta_{CRS}+\left(N_{t}+K\cdot \psi \right)}{\eta_{RB_{tot}}N_{g}}\right)>0.5\\ 0.5\cdot K^{\prime }\cdot \left[\log_{2}\left(1+\frac{N_{t}^{\prime }\hat{\xi }\left(1-\frac{N_{g}^{\prime }}{\varepsilon }\right)\rho_{r}}{K^{\prime }\left(\rho_{r}+1\right)}\right)\right],\;\left(1-\frac{\eta_{CRS}+\left(N_{t}+K\cdot \psi \right)}{\eta_{RB_{tot}}N_{g}}\right)\leq 0.5 \end{array}\right.$$

where $K^{\prime }$, $N_{t}^{\prime }$, and $N_{g}^{\prime }$ are corresponding *K*, $N_{t}$, and $N_{g}$ when $\left(1-\frac{\eta_{CRS}+\left(N_{t}+K\cdot \psi \right)}{\eta_{RB_{tot}}N_{g}}\right)=0.5$.

A few observations are in order.

*Observation* *1:* As $N_{g}$ increases, bandwidth increases due to RS overhead reduction.
*Observation* *2:* As $N_{g}$ increases, SINR decreases due to channel estimation error.

Increasing $N_{g}$ is beneficial to the system when system is working in bandwidth limited regime, but if system is working in power limited regime and/or interference limited regime, increasing $N_{g}$ worsens system performance.

Two strategies can be considered to choose $N_{g}$. First, $N_{g}$ can be determined to maximize SE which can be represented as follows for ZF precoding and MF precoding respectively.

(30) $$\begin{array}{cc} \max_{N_{g}} & \left(\Theta \cdot \sum_{k=1}^{K}E\left[\log_{2}\left(1+\frac{\left(N_{t}-K\right)\hat{\xi }\left(1-\frac{N_{g}}{\varepsilon }\right)\rho_{r}}{K\left(\left(1-\hat{\xi }\left(1-\frac{N_{g}}{\varepsilon }\right)\right)\rho_{r}+1\right)}\right)\right]\right)\\ s.t. & \;\Theta \geq 0.5\\ & \;\rho_{r}\leq \rho_{r,\max}, \end{array}$$

(31) $$\begin{array}{cc} \max_{N_{g}} & \left(\Theta \cdot \sum_{k=1}^{K}E\left[\log_{2}\left(1+\frac{N_{t}\hat{\xi }\left(1-\frac{N_{g}}{\varepsilon }\right)\rho_{r}}{K(\rho_{r}+1)}\right)\right]\right)\\ s.t. & \;\Theta \geq 0.5\\ & \;\rho_{r}\leq \rho_{r,\max}, \end{array}$$

where $\Theta =\left(1-\frac{\eta_{CRS}+\left(N_{t}+K\cdot \psi \right)}{\eta_{RB_{tot}}N_{g}}\right)$ and $\rho_{r,\max}$ is the maximum SNR which reflect the maximum allowable TX power. This is a typical setting for wireless communication systems up to now. However, to support distributed sensors and/or sensor networks, energy efficiency can be more important than SE depending on applications. In a certain application, sensor devices do not require high data rate, but maintaining moderate data rate can be enough. In this situation, $N_{g}$ can be determined based on the minimum required SE in a given circumstances. In particular, for the energy efficient sensor networks, if the minimum required SE is satisfied, the system can operates efficiently with high energy efficiency and a moderate data rate. Also, if determination of $N_{g}$ causes lower SE than the requirement, it could be problematic to the proper operation of sensor networks. It is particularly important to maintain the required data rate for industrial IoT and/or sensor networks. If $N_{g}$ can be derived as a closed-form equation, it can be very useful to reduce the computational complexity and delay of the system. The inverses of Equations (28) and(29) can provide $N_{g}$ with given required SE, however the exact closed-form inverse equations of Equations (28) and(29) are impossible, because $N_{g}$ terms exist both inside and outside of log term. Instead, by keeping the $\frac{\eta_{CRS}+\left(N_{t}+K\cdot \psi \right)}{\eta_{RB_{tot}}N_{g}}$ as 0.5, the minimum value of data portion, we can get $N_{g}$ with satisfying the minimum required value of SE. The proposed scheme is can be established based on following Proposition.

**Proposition** **1.** *$\left(1-\frac{\eta_{CRS}+\left(N_{t}+K\cdot \psi \right)}{\eta_{RB_{tot}}N_{g}}\right)\cdot K\cdot \left[\log_{2}\left(1+\frac{\left(N_{t}-K\right)\hat{\xi }\left(1-\frac{N_{g}}{\varepsilon }\right)\rho_{r}}{K\left(\left(1-\hat{\xi }\left(1-\frac{N_{g}}{\varepsilon }\right)\right)\rho_{r}+1\right)}\right)\right]\geq \Delta \cdot K\cdot \left[\log_{2}\left(1+\frac{\left(N_{t}-K\right)\hat{\xi }\left(1-\frac{N_{g}}{\varepsilon }\right)\rho_{r}}{K\left(\left(1-\hat{\xi }\left(1-\frac{N_{g}}{\varepsilon }\right)\right)\rho_{r}+1\right)}\right)\right]$ iff Δ≤0.5 and $\left(1-\frac{\eta_{CRS}+\left(N_{t}+K\cdot \psi \right)}{\eta_{RB_{tot}}N_{g}}\right)\geq 0.5$*.

**Proof.**  Since $\left(1-\frac{\eta_{CRS}+\left(N_{t}+K\cdot \psi \right)}{\eta_{RB_{tot}}N_{g}}\right)\geq 0.5$, if Δ≤0.5, then

$$\frac{\left(1-\frac{\eta_{CRS}+\left(N_{t}+K\cdot \psi \right)}{\eta_{RB_{tot}}N_{g}}\right)\cdot K\cdot \left[\log_{2}\left(1+\frac{\left(N_{t}-K\right)\hat{\xi }\left(1-\frac{N_{g}}{\varepsilon }\right)\rho_{r}}{K\left(\left(1-\hat{\xi }\left(1-\frac{N_{g}}{\varepsilon }\right)\right)\rho_{r}+1\right)}\right)\right]}{\Delta \cdot K\cdot \left[\log_{2}\left(1+\frac{\left(N_{t}-K\right)\hat{\xi }\left(1-\frac{N_{g}}{\varepsilon }\right)\rho_{r}}{K\left(\left(1-\hat{\xi }\left(1-\frac{N_{g}}{\varepsilon }\right)\right)\rho_{r}+1\right)}\right)\right]}=\frac{\left(1-\frac{\eta_{CRS}+\left(N_{t}+K\cdot \psi \right)}{\eta_{RB_{tot}}N_{g}}\right)}{\Delta }\geq 1,$$

thus Proposition 1 is satisfied. ☐

From Proposition 1, the maximum value of Δ is 0.5. This means, if we fix Θ=0.5 and derive the $N_{g}$, the minimum required SE can be satisfied. Proposition 1 also can be applied to the MF precoding case.

**Corollary** **1.** *$\left(1-\frac{\eta_{CRS}+\left(N_{t}+K\cdot \psi \right)}{\eta_{RB_{tot}}N_{g}}\right)\cdot K\cdot \left[\log_{2}\left(1+\frac{N_{t}\hat{\xi }\left(1-\frac{N_{g}}{\varepsilon }\right)\rho_{r}}{K(\rho_{r}+1)}\right)\right]\geq \Delta \cdot K\cdot \left[\log_{2}\left(1+\frac{N_{t}\hat{\xi }\left(1-\frac{N_{g}}{\varepsilon }\right)\rho_{r}}{K(\rho_{r}+1)}\right)\right]$ iff Δ≤0.5 and $\left(1-\frac{\eta_{CRS}+\left(N_{t}+K\cdot \psi \right)}{\eta_{RB_{tot}}N_{g}}\right)\geq 0.5$*.

**Proof.**  Proof of Corollary 1 can follow the same way of that of Proposition 1. ☐

Let $\left(1-\frac{\eta_{CRS}+\left(N_{t}+K\cdot \psi \right)}{\eta_{RB_{tot}}N_{g}}\right)=\delta$, then the closed-form equation for $N_{g}$ for ZF precoding and MF precoding with the minimum required $SE_{req}$ can be represented as follows:

(32) $$N_{g,ZF}\to \frac{\varepsilon \left(K+K\rho_{r}-2K\rho_{r}\hat{\xi }+N_{t}\rho_{r}\hat{\xi }-K2^{\left(\frac{{\tilde{SE}}_{ZF}}{\delta K}\right)}-K\rho_{r}2^{\left(\frac{{\tilde{SE}}_{ZF}}{\delta K}\right)}+K\rho_{r}\hat{\xi }2^{\left(\frac{{\tilde{SE}}_{ZF}}{\delta K}\right)}\right)}{\rho_{r}\hat{\xi }\left(-2K+N_{t}+K2^{\left(\frac{{\tilde{SE}}_{ZF}}{\delta K}\right)}\right)}.$$

(33) $$N_{g,MF}\to \frac{\varepsilon K+\varepsilon K\rho_{r}+\varepsilon N_{t}\rho_{r}\hat{\xi }-\varepsilon K2^{\left(\frac{{\tilde{SE}}_{MF}}{\delta K}\right)}-\varepsilon K\rho_{r}2^{\left(\frac{{\tilde{SE}}_{MF}}{\delta K}\right)}}{N_{t}\rho_{r}\hat{\xi }}.$$

where ${\tilde{SE}}_{ZF}$ and ${\tilde{SE}}_{MF}$ are required minimum SE for ZF and MF precoded systems. By fixing δ=0.5, the lowest bound, we can get $N_{g}$ that satisfy to ${\tilde{SE}}_{ZF}$ and ${\tilde{SE}}_{MF}$ with some margin.

**Figure 3:**
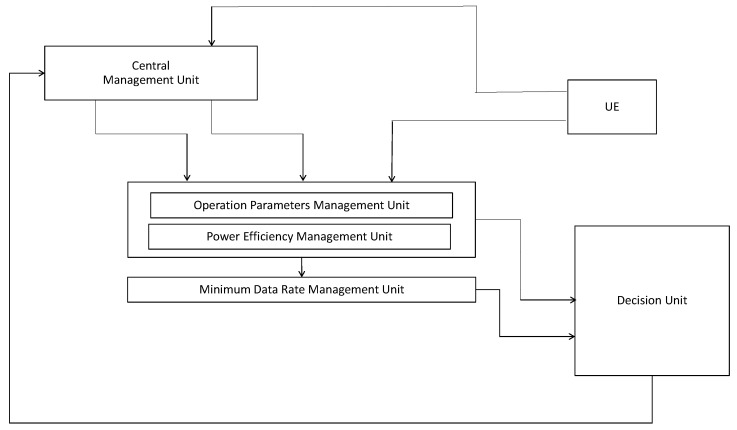
Example of system block diagram for proposed scheme.

The proposed scheme can be used in various ways. In particular, it would be very effective to reduce the power consumption of massive MIMO-based wireless sensor networks. Figure 3 shows example of a system block diagram for the proposed scheme. It consists of several system blocks.

First, the central management unit can decide the power consumption and/or power efficiency of the system. Then, BS measures current power efficiency. BS also has various operation parameters, such as $N_{t}$, *K*, *B*, and $\rho_{t}$ and so on. Based on the power efficiency and operation parameters, the minimum data rate that must be satisfied for the system can be derived. If the system is designed for for Industrial internet, there would be a threshold for data rate, and the system should set the minimum data rate higher than the threshold. Then, from the minimum data rate and other parameters that are necessary, $N_{g}$ can be determined. The BS can receive feedback from UEs to check if they need a higher data rate depending on the situation. Wireless channel is not stable, so there could be several situations that can arise. Then, the procedures we mentioned can be repeated for a given number of iterations. There could be an interval for the determination of $N_{g}$. If the interval is small, then the performance would be better, but complexity could be a problem or vice versa.

**Figure 4:**
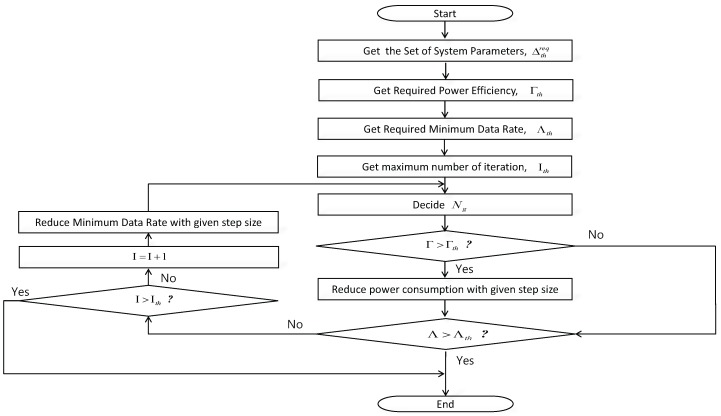
Example of operation flow chart.

Example of operation flow chart is presented in Figure 4. First, BS can get system parameters, then it gets required power efficiency. Based on system parameters and power efficiency, the required minimum data rate can be derived. The $N_{g}$ can be derived using the proposed closed-form equation. The RS can be designed and used based on $N_{g}$. Then, communications between massive MIMO and distributed IoT devices would be conducted. If power consumption is higher than the threshold, we can reduce power consumption by any kind of parameter adjustment. Typically, TX power consumes a lot of power, thus it can be reduced to increase power efficiency. However, TX power reduction accompanies data rate reduction. If the data rate is higher than threshold even though TX power is reduced, then system is in good shape and the procedure can be finished, but if data rate is lower than the threshold due to the TX power reduction, then in a given number of iterations, we should reduce required minimum data rate to satisfy power efficiency. The determination of thresholds for the number of iterations, power consumptions, data rate are the system designer’s choice. For the better understand and implementation, we provide the detailed algorithm as pseudo-code type in Algorithm 1.

The required number of iterations for the system can be changed based on system parameters. There are several parameters including the threshold for the number of iterations, thus system designer can decide it between performance and delay/complexity trade-off.

**Algorithm 1:** Operation of proposed scheme
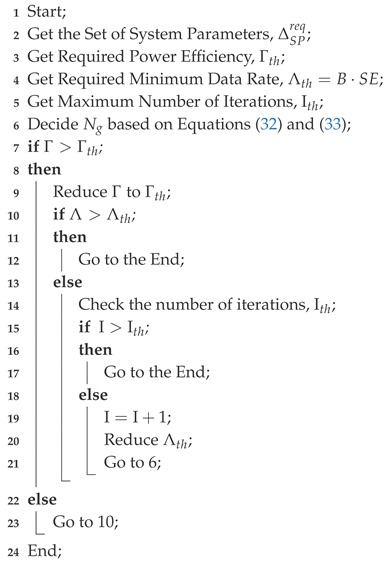


## 5. Numerical Results and Discussion

In this Section, we verify the proposed scheme using monte-carlo (MC) simulations with $10^{4}$ iterations, and present the related discussions. The simulation parameter for this section is shown in Table 3.

We used $K=0.1N_{t}$ to meet the minimum requirement of channel hardening effect. The noise power $N_{0}B$ is normalized to unity with $\rho_{t}=$ 80 W and B= 20 MHz bandwidth. Assuming we use a macro-cell type setup with 2GHz carrier frequency, the path loss in dB is modeled as 128.1 + 37.6 log(*d*) with distance *d* in kilometers [41]. Then, the setup is equivalent to average user distance of 1.194 km. We set $\hat{\xi }=1$ and ϵ=300 in Equation (12). Obviously, the parameter can be changed depending on the applied technique and circumstances.

**Figure 5:**
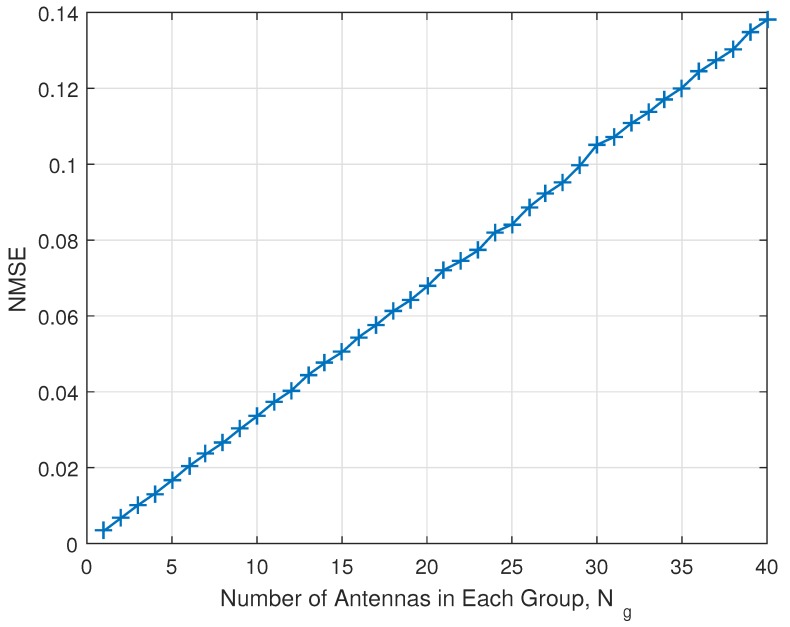
Seriousness of channel estimation error: normalized mean square error (NMSE) versus $N_{g}$.

Before showing the numerical results, we present the seriousness of channel estimation error in Figure 5.

We show the seriousness of channel estimation error using normalized mean square error (NMSE) which is defined as:

(34) $$NMSE=\frac{{∥H-\hat{H}∥}_{2}^{2}}{{∥H∥}_{2}^{2}}.$$

$\hat{H}$ is the estimated channel and it is a function of $N_{g}$ as we shown in the previous section. NMSE is linearly increased as $N_{g}$ increases. When $N_{g}$ is 40, NMSE has reached around 14%, while we can get bandwidth benefit thanks to the RS overhead reduction.

**Figure 6:**
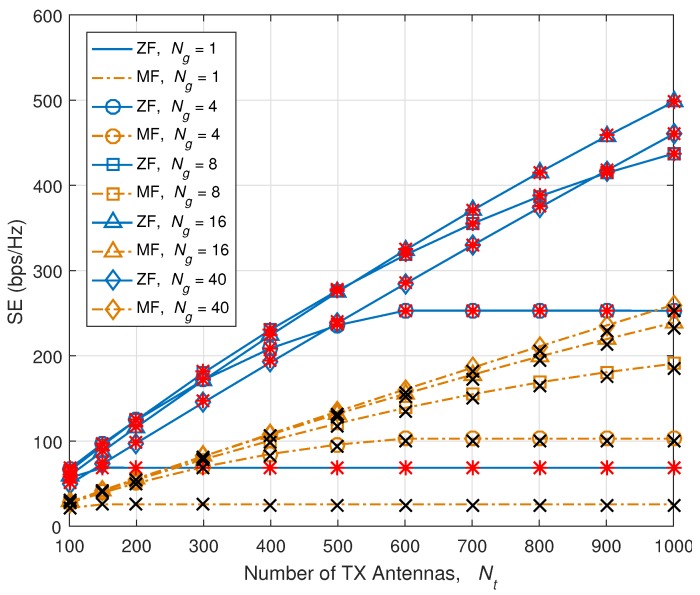
SE versus number of TX antennas, $N_{t}$. Red ‘*’s and black ‘x’ indicate the simulation results.

Figure 6 shows the numerical results of SE versus $N_{t}$. Red ‘*’s and black ‘x’ indicate the simulation results, and lines are plotted based on theoretical analysis. Our theoretical analysis is well-matched with the simulation result. When $N_{g}$ is small which is less than 4, due to the excessive RS overhead which reaches the maximum allowable RS overhead, SE is not increased, even though $N_{t}$ is increased. However, when $N_{g}$ is larger than 8, the SE increases, as $N_{t}$ increases up to 1000. RS overhead reduction technique is definitely necessary to increase $N_{t}$ and SE, and fully enjoy the benefit of massive MIMO. However, increasing $N_{g}$ causes channel estimation error as we have seen from previous Section. Increasing $N_{g}$ gives a bandwidth advantage by giving more room for data, but due to channel estimation error, SINR becomes worse.

**Figure 7:**
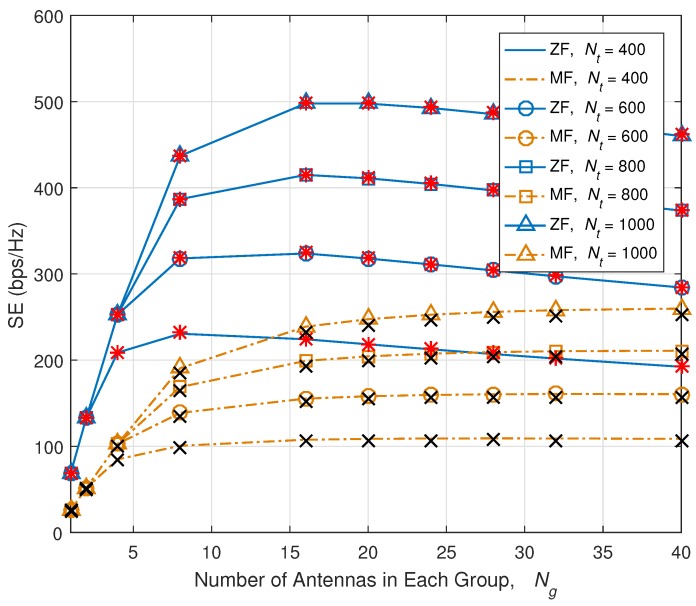
SE versus number of antennas in each group, $N_{g}$. Red ‘*’s and black ‘x’ indicate the simulation results.

To show the relationship between SE and $N_{g}$, we present Figure 7, SE versus $N_{g}$. As observed, if $N_{g}$ is larger than 16, the SE is reduced due to worsened SINR. It is noticeable in ZF precoding because as $N_{g}$ grows, IUI terms grows. The IUI term in ZF precoding is almost negligible when there is little channel estimation error. In the case of MF precoding, there is no SE reduction, but SE is fixed to a certain point even though $N_{g}$ increases. This is because IUI is already a dominant term in MF precoding even when there is little channel estimation error. When we use MF precoding, if $N_{g}$ is larger than 16, the system is already working in a power limited regime, thus increasing $N_{g}$ gives little help in increasing SE. This phenomenon is maintained even if we increase $N_{t}$.

**Figure 8:**
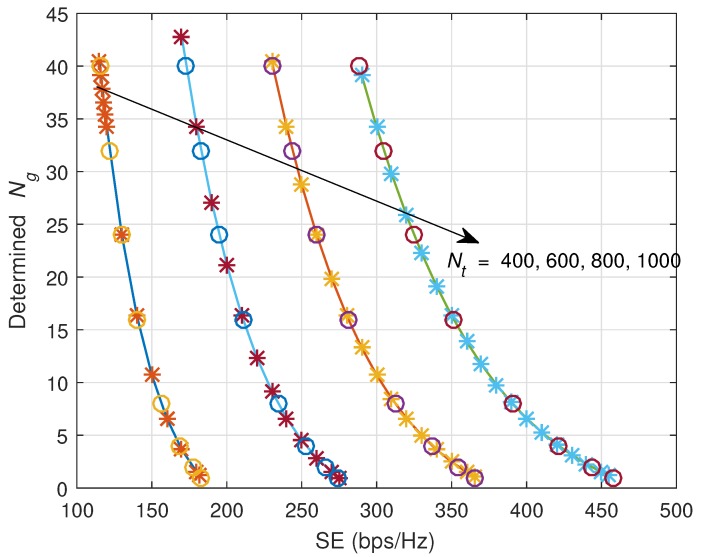
Determined $N_{g}$ versus guaranteed SE (bps/Hz) when ZF precoding is applied. ‘*’ marks indicate the results of closed-form SE equation, and ‘O’ marks indicate results of MC simulations.

**Figure 9:**
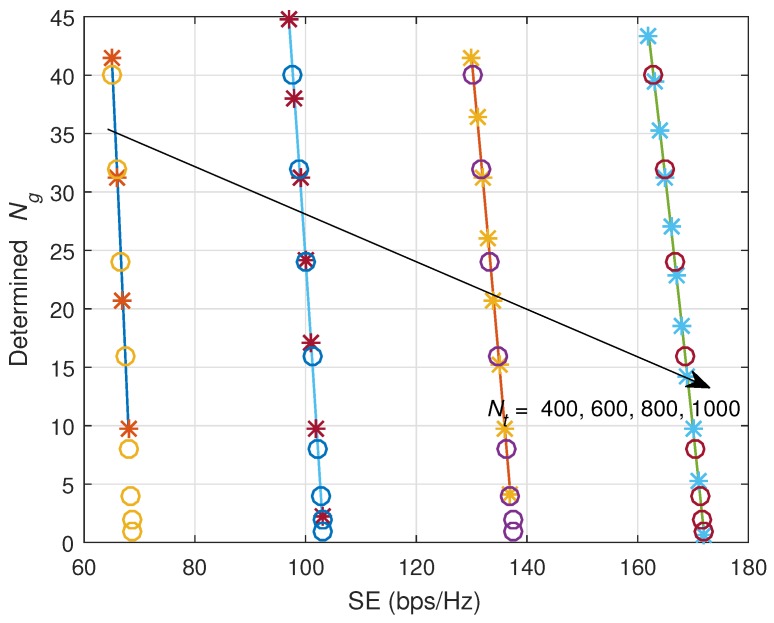
Determined $N_{g}$ versus guaranteed SE (bps/Hz) when MF precoding is applied. ‘*’ marks indicate results of closed-form SE equation, and ‘O’ marks indicate results of MC simulations.

Now, we show the effectiveness of the closed-form equation for $N_{g}$ determination. Figure 8 and Figure 9 show the determined $N_{g}$ versus SE. ‘*’ marks indicate results of closed-form SE equation, and ‘x’ marks indicate results of MC simulations. Lines are plotted based on theoretical derivations. As observed, all 3 kinds of results are well-matched, and the derived closed-form equations for $N_{g}$ can be used with high accuracy. In the case of ZF precoding, the lower bound of SE is sensitive to the variation of $N_{g}$, while in the case of MF precoding, the lower bound of SE is not so sensitive to the variation of $N_{g}$.

Maintaining SE lower bounds is quite important to the energy efficient sensor network. The important metric of wireless sensor network is moving from SE to energy efficiency. However, keeping a stable SE lower bound is also very important for some kinds of sensor network, such as Industrial internet network. The proposed scheme provides the logic of keeping high energy efficiency maintaining SE lower bound, thus it can be a core technology for the massive MIMO-based energy efficient sensor network.

**Figure 10:**
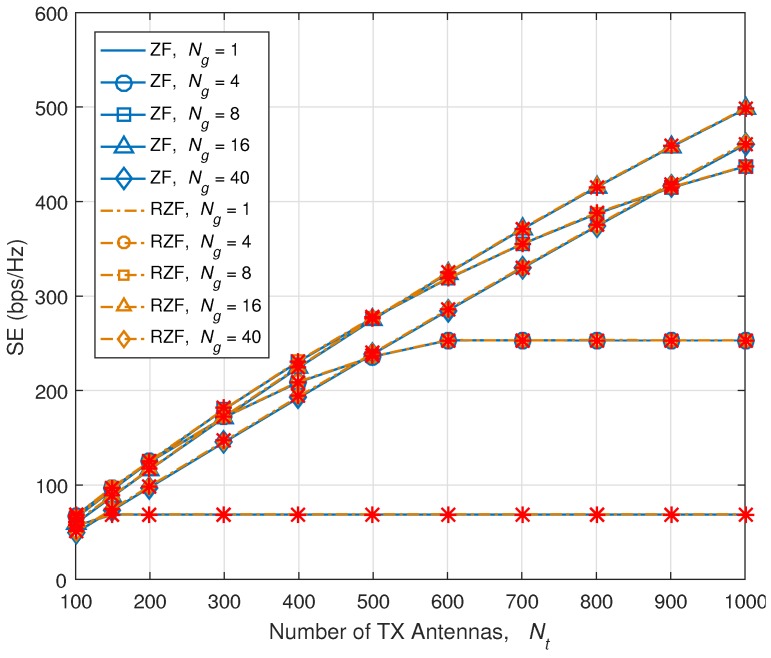
SE comparison of ZF and RZF precoding. ‘*’ marks indicate results of closed-form SE equation for ZF precoding.

As we mentioned in the previous section, for the case of RZF precoding, we can use the case of ZF precoding. Figure 10 presents the SE comparison of ZF precoding and RZF precoding. We use $\nu ≜diag\left[\frac{1}{\rho_{r}},\cdots ,\frac{1}{\rho_{r}}\right]$ which is the downlink RX inverse SNR matrix [28,30,40]. As observed, the SE of RZF precoding also exactly matches with the analytical SE of ZF precoding. Thus, all the analysis for ZF precoding in this paper is also applicable to RZF.

To completely validate the proposed system, it is important to perform real measurement. Since we show the feasibility of the proposed system in this paper, the real measurement for the system can be performed in future work.

## 6. Conclusions

In this paper, we have proposed a design logic of RS overhead reduced massive MIMO for energy efficient wireless sensor networks. The contribution of this paper can be divided into two parts. First, we have presented the closed-form achievable SE of massive MIMO with channel estimation error. Second, based on the the results, we have provided an effective scheme to determine the number of antennas required in each group. Numerical results showed that the derived theoretical achievable SE and $N_{g}$ are accurate enough, and thus be provided as an useful tool for the design of massive MIMO-based wireless sensor networks and many other related engineering applications.

## Figures and Tables

**Table 1:** The precoding matrices of MF (matched filtering), ZF (zero-forcing), and RZF.

Precoder	MF	ZF	RZF
F	$H^{H}$	$H^{H}{\left(HH^{H}\right)}^{-1}$	$H^{H}{\left(HH^{H}+\nu I_{K}\right)}^{-1}$

Where superscript “H” denotes conjugate transpose, ${\left(\cdot \right)}^{-1}$ is the inverse operator, and $I_{K}$ is the K×K identity matrix.

**Table 2:** Summary of approximated SINRs.

$\gamma_{k,\mathrm{MF}}^{\mathrm{ref}}$	$\gamma_{k,\mathrm{ZF}}^{\mathrm{ref}}$	${\hat{\gamma }}_{k,\mathrm{MF}}$	${\hat{\gamma }}_{k,\mathrm{ZF}}$
$\frac{N_{t}}{K}\left(\frac{\rho_{r}}{\rho_{r}+1}\right)$	$\frac{N_{t}-K}{K}\left(\rho_{r}\right)$	$\frac{N_{t}}{K}\left(\frac{\xi_{}^{2}\rho }{\rho_{r}+1}\right)$	$\frac{N_{t}-K}{K}\left(\frac{\xi^{2}\rho_{r}}{\left(1-\xi^{2}\right)\rho_{r}+1}\right)$

**Table 3:** Simulation Parameters.

Parameter	Value
Coherence Time, $\tau_{c}$	5 ms
Coherence Bandwidth, $BW_{c}$	180 kHz
max RS time, $\tau_{p}$	2.5 ms
Total symbols in coherence interval, $S_{tot}$	840
TX power, $\rho_{t}$	80 W
Signal Bandwidth, *B*	20 MHz
Carrier Frequency, $f_{c}$	2 GHz
Path Loss Model	ETSI
Number of TX antennas, $N_{t}$	100∼1000
Max. number of serviced UEs for downlink,	100
Error factor without RS overhead reduction, $\hat{\xi }$	1
Precoding	ZF, MF
